# Association between knee angles at initial contact and post-landing knee ranges of motion in athletes with and without anterior cruciate ligament reconstruction

**DOI:** 10.1038/s41598-026-41776-w

**Published:** 2026-03-03

**Authors:** Andrea Baldazzi, Lorenzo Rum, Riccardo Borzuola, Corentin Bosio, Hélène Pillet, Fabrizio Margheritini, Elena Bergamini

**Affiliations:** 1 Department of Movement, Human and Health Sciences, University of Rome “Foro Italico”, Rome, Italy; 2 Department of Human Sciences, Link Campus University, Rome, Italy; 3 Arts et métiers ParisTech, Université Sorbonne Paris Nord, Paris, France; 4 Arts et Métiers Sciences and Technologies, Institut de Biomécanique Humaine Georges Charpak, Paris, France; 5 Department of Management, Information and Production Engineering, University of Bergamo, Bergamo, Italy

**Keywords:** Knee kinematics, Range of motion, Return to sport, Anterior cruciate ligament injury, Soccer, Biomedical engineering, Rehabilitation

## Abstract

Altered knee kinematics is associated with Anterior Cruciate Ligament (ACL) injury risk. Knee angle at initial contact (A_IC_) and range of motion (RoM) after landing are often used interchangeably as ACL risk indicators, yet their relationship remains unclear. Moreover, no consensus exists about the time window to be considered for the RoM. This study explored the degree of association between knee A_IC_ and two RoMs (unstandardized: from IC to maximum knee flexion angle, ARoM_FULL_; standardized: within 100 milliseconds after IC, defining a Risk Time Window for ACL injury, ARoM_RTW_). Eleven ACL-reconstructed soccer players and 20 healthy controls performed Single Leg Hop (SLH) and Single Leg Cross Drop Landing (SLCDL) tasks on a force plate. Knee kinematics was recorded in the sagittal, frontal, and transverse planes. Correlation analysis showed moderate-to-very-high positive correlations between ARoM_FULL_ and ARoM_RTW_ across all tasks, limbs, and planes (r: 0.56–0.99), supporting their similarity in capturing early post-landing motion. Conversely, significant correlations between A_IC_ and both RoMs were only found in frontal plane (r: 0.48–0.69), suggesting that A_IC_ alone may not reliably reflect knee dynamics after impact. Since ACL injuries typically occur within 100 ms post-landing, ARoM_RTW_ may offer a practical metric for injury screening and rehabilitation monitoring.

## Introduction

The Anterior Cruciate Ligament (ACL) plays a key role in stabilizing the knee during dynamic high-impact activities involving different planes of motion^1^. For this reason, its rupture is considered among the most critical injuries in sports^2^. ACL injury also presents a high rate of re-rupture (up to 29.5%), both regarding the reconstructed and the contralateral limb^3^. This is mainly due to the multifactorial nature of this lesion, with several internal and external risk factors interacting and influencing each other, such as congenital aspects (anatomical, hormonal, neuromuscular) and/or the unpredictability of the sports context^4,5^. Consequently, improving our understanding of the biomechanical mechanisms underlying ACL injury risk is essential to inform preventive strategies and optimize rehabilitation protocols.

A large number of studies have focused on the analysis of the injury mechanisms, thus allowing the identification of critical patterns that increase the risk of ACL rupture. In the sagittal plane, higher stresses are reported when the knee joint is slightly flexed, from 5° up to 30°, during load acceptance^6,7^. In the frontal plane, numerous works have pointed out how valgus trends are the most common non-physiological and harmful knee movements during high-intensity activities, greatly increasing the risk of ACL rupture^7–9^. These trends are often associated with abnormal movements of the tibiofemoral joint on the transverse plane^10^. On this basis, one of the most common mechanisms dealing to ACL rupture is the co-occurrence of slightly flexed knee, valgus collapse and abnormal rotation of the tibia with respect to the femur, as confirmed by studies involving video analysis that evaluated the mechanism of ACL injury during competitions (e.g., in soccer, basketball, handball, rugby)^11–13^. In addition, the existing literature agrees that rupture occurs within the first 100 milliseconds following foot contact with the ground^14,15^, as the highest stresses to the knee occur within this narrow risk time window (RTW)^16,17^. These findings underscore the importance of precisely quantifying lower limb kinematics in order to enhance risk assessment and refine injury prevention models^18,19^.

In this context, the possibility to accurately and reliably track the athletes’ movements is of the utmost importance. Marker-based optoelectronic systems combined with force platforms represent the gold-standard to obtain objective and reliable values when investigating kinematic and dynamic parameters of interest for ACL rupture prevention and recovery^20,21^. Among those, lower limb joint kinematics has been broadly investigated in the literature^22–25^. Indeed, numerous 3D-based studies have revealed altered kinematics in individuals who underwent ACL surgery compared with healthy athletes^26–28^. These movement patterns reflect ongoing neuromuscular control issues and exhibit joint angles close to those involved in the injury mechanisms^28–30^.

Parameters obtained from the knee joint angular kinematics, including peak angles, angle at initial contact (IC), and joint range of motion (RoM), have been associated with ACL injury mechanisms, being widely used over the past years as risk indicators^18,31–33^. In particular, increased frontal plane knee angles at IC during drop vertical jump have been linked to a higher likelihood of ACL injury, with recent evidence showing significantly greater valgus angles at IC in female athletes who incurred ACL injury^34^. Similarly, greater frontal plane RoMs during drop jump (from IC to toe-off) have been reported in female athletes who later sustained an ACL tear^18^.

Despite the relevance of these metrics, the relationship between the knee angle at IC and subsequent post-landing RoM remains unclear, limiting their precise application as ACL injury predictors^15,35^. While the knee angle at IC refers to a discrete instant of time, RoM following ground contact reflects the knee angular displacement over a defined time window. From a biomechanical perspective, these variables are sequential in nature, as RoM after landing inherently follows IC due to foot-ground interaction. However, it is still uncertain whether the post-landing RoM is influenced by the knee angle at IC or mainly reflects independent neuromuscular control strategies^35^. If a meaningful association exists, specific knee angles at IC could be expected to correspond to characteristic post-landing RoM patterns; however, this hypothesis has not yet been systematically investigated.

Moreover, the literature provides no clear indication regarding the optimal time window duration for assessing the knee joint RoM after landing, particularly in relation to the temporal characteristics of ACL injury mechanisms^14,15^. The standardization of the time window for RoM assessment represents a relevant methodological issue, as it would enable more meaningful comparisons across studies. However, it remains unclear whether RoM values derived from different time windows (e.g., a standardized 100-ms RTW versus the time to post-landing peak RoM) reflect distinct or overlapping aspects of post-landing knee kinematics. Demonstrating a strong correlation between RoM measures computed over different temporal intervals would suggest that these metrics capture similar features of post-landing knee behavior, thereby supporting the feasibility of adopting a standardized 100-ms risk time window.

The aim of the present work is thus to explore the relationship between the knee angle at IC and the knee RoM (after IC) in patients with ACL reconstruction and in healthy participants. To this aim, the knee angular displacement in the three anatomical planes was obtained during two landing motor tasks. Knee angles at IC and knee RoM considering two different time windows (from IC to the instant of maximum knee flexion and the first 100 milliseconds after IC, i.e., the RTW) were extracted and their relationship was tested through correlation analysis. Our first hypothesis was that the knee RoMs evaluated in these two time-windows would be highly correlated, indicating that these two parameters are similarly informative in depicting the knee joint behavior immediately after landing. Our second hypothesis was that the angle at IC and both RoMs would not necessarily be correlated. This would indicate that knee RoMs follow a specific pattern independent of the knee angle at IC, suggesting caution in adopting these two metrics interchangeably as ACL injury risk predictor.

## Materials and methods

### Participants

Eleven soccer players with ACL reconstruction (age: 24.3 ± 5.8 years; body mass: 82.2 ± 8.8 kg; stature: 1.83 ± 0.07 m; time post-surgery: 0.97 ± 0.2 years) (ACLR) and twenty healthy controls (age: 25.0 ± 4.4 years; body mass: 72.9 ± 6.1 kg; stature: 1.79 ± 0.06 m) (HC) participated in the study. The sample size was determined using the G*Power software (ver. 3.1.9.4; Heinrich-Heine-Universität Düsseldorf, Düsseldorf, Germany) through an a priori power analysis based on a bivariate correlation test (two-tailed, significance level of 0.05, power of 0.8, and correlation coefficient of 0 assumed for the null hypothesis). Based on the initial hypothesis of moderate to strong correlation, a correlation coefficient of 0.75 was assumed for hypothesis testing, with a resulting required sample size of 11 and a critical r value of ± 0.60.

All tested participants were recruited by personal contacts of the authors and/or through a database provided by an orthopedic surgeon within the working group, over a period from April 2022 to May 2024. Subjects attended the experimental session only once. All athletes were between 18 and 40 years old, with a Tegner scale score greater than 6, corresponding to good levels of physical activity^36^. For ACLR, inclusion criteria were: *(i)* a post-operative period from ACL surgery ranging from 8 to 12 months (intended as the conventional period of return to sport, RTS); *(ii)* no history of additional knee surgeries in the past; *(iii)* clearance to RTS through specialist’s validation; *(iv)* no physiological RoM limitations at the knee joint. In addition, all participants with ACLR were required to confirm, prior to recruitment, that they had returned to full participation in sporting activities, by answering the following questions: *(a)* "Are you currently participating in sports activities without any type of restriction, both with regard to training and official competitions?"; *(b)* "Since you returned to sport, have there been any occasions that required you to stop because of your knee?". For HC, inclusion criteria were the total absence of significant lower limb injuries that could require surgery or causing ongoing disability. For analysis purposes, the dominant lower limb was identified in HC group as the limb that was used to shoot a ball on a target, namely the one with which they usually touched the ball during the game^37,38^. Before starting data acquisition, written informed consent was obtained from each participant. The study procedures were performed in accordance with the principles of the Declaration of Helsinki and approved by the institutional review board of the University of Rome “Foro Italico” (CAR 118/2022).

### Instruments

The 3D trajectories of 52 reflective markers were measured by a 9-camera stereophotogrammetric system (Vicon Nexus, Oxford, UK, 200 Hz). Markers were placed from the same operator on anatomical landmarks following a full-body protocol proposed by Pillet et colleagues, largely focused on lower limb analysis^39^. Three force plates (one 0.9 × 0.6 m, two 0.6 × 0.6 m, AMTI, Watertown, Massachusetts, USA, 1000 Hz) were used to collect ground reaction forces (GRF), which were then used to define the instant of IC, defined as the instant of time when the vertical GRF exceeded 20 N^40,41^.

### Experimental protocol

**Fig. 1:**
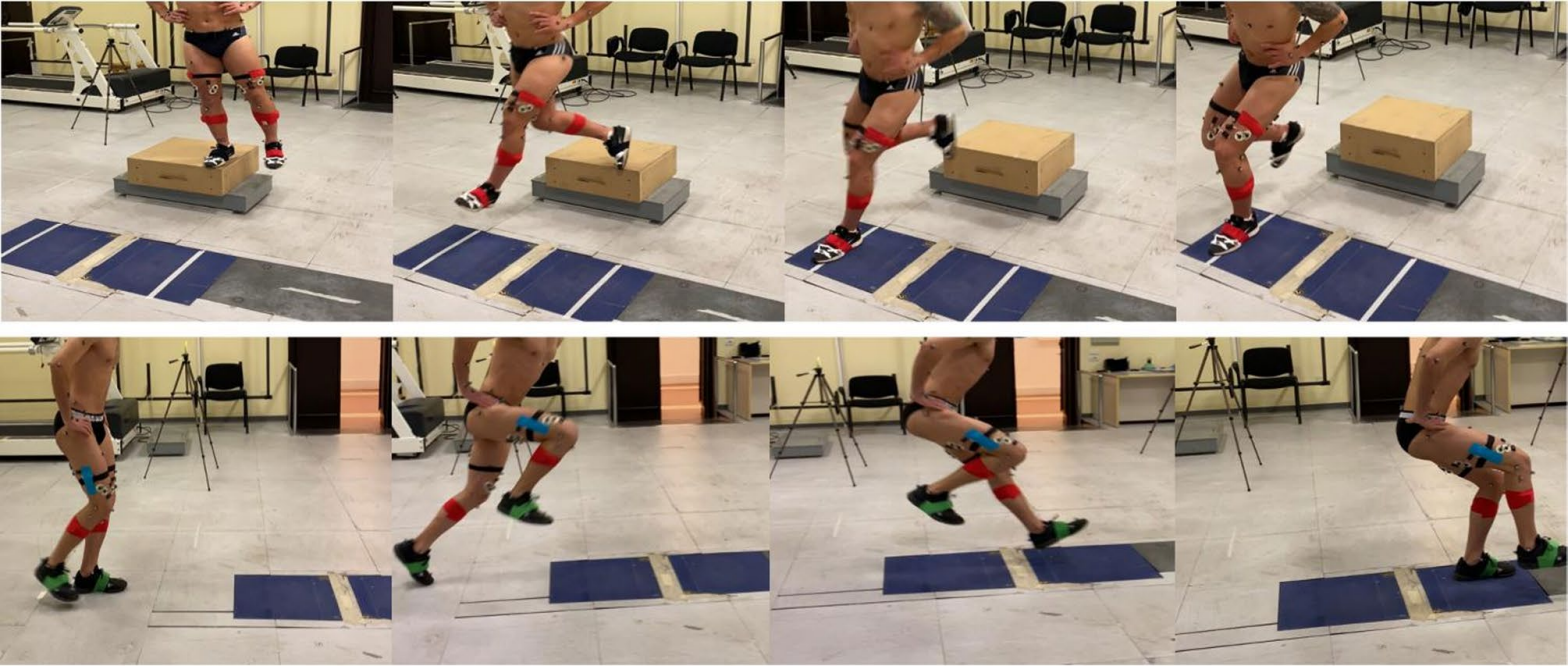
Phase sequence during Single Leg Cross Drop Landing (SLCDL, above) and Single Leg Hop (SLH, below) execution.

After placing the markers on the skin at the respective anatomical landmarks, a standardized 5-minute warm-up on a treadmill was completed by each participant. After the warm-up, participants were instructed and provided with a short familiarization period to practice the test execution. Each participant performed two functional tests involving multiplanar single-leg closed-chain movements typical of open-skill sports practice: the Single Leg Hop (SLH) and the Single Leg Cross Drop Landing (SLCDL) tests (Fig. 1). In the SLH test, participants were asked to perform a single-leg jump with the aim of reaching the largest distance possible and maintaining a stable position upon landing. Before the propulsion phase, free swinging of the non-tested limb was allowed^42^. In the SLCDL test, participants performed a single-leg cross landing from a 31 cm-high box, which was positioned 40 cm away from the force plates. When hopping off the box, the participants were instructed to avoid jumping movements and to cross over with the landing foot, reaching the opposite (farther) side of the force plate^43^. Trials presenting an upward motion of the subject during the SLCDL task were removed following a real-time verification of the trajectory of the pelvis marker. For both tests, participants were asked to keep their hands on the iliac crests to avoid the interference of the arms in the movement execution. Tests were considered valid if participants were able to maintain a stable monopodalic balance for at least 2 s after landing on the force plate, avoiding additional hops or foot adjustments following ground contact^42^. Participants wore their own sport shoes, corresponding to those commonly used for their off-field training. Each test was performed three times per limb which were defined as: Reconstructed (R_L_) / Uninvolved (U_L_) for ACLR; Non-Dominant (ND_L_) / Dominant (D_L_) for HC. The order of the tests was randomly selected.

### Data processing

The present study focuses exclusively on the landing phase of both motor tasks. For both limbs of each participant, the trial with the highest value of vertical GRF was conservatively selected for the following analysis. This is motivated by the premise that higher vertical GRF values are one of the main indicators of increased joint loads/decreased shock absorption during landing, turning into subsequent greater stress on the ACL^20^. Therefore, we decided to select trials presenting the highest stress and potential risk for the knee joint based on this parameter to align with the purposes of this study.

**Fig. 2:**
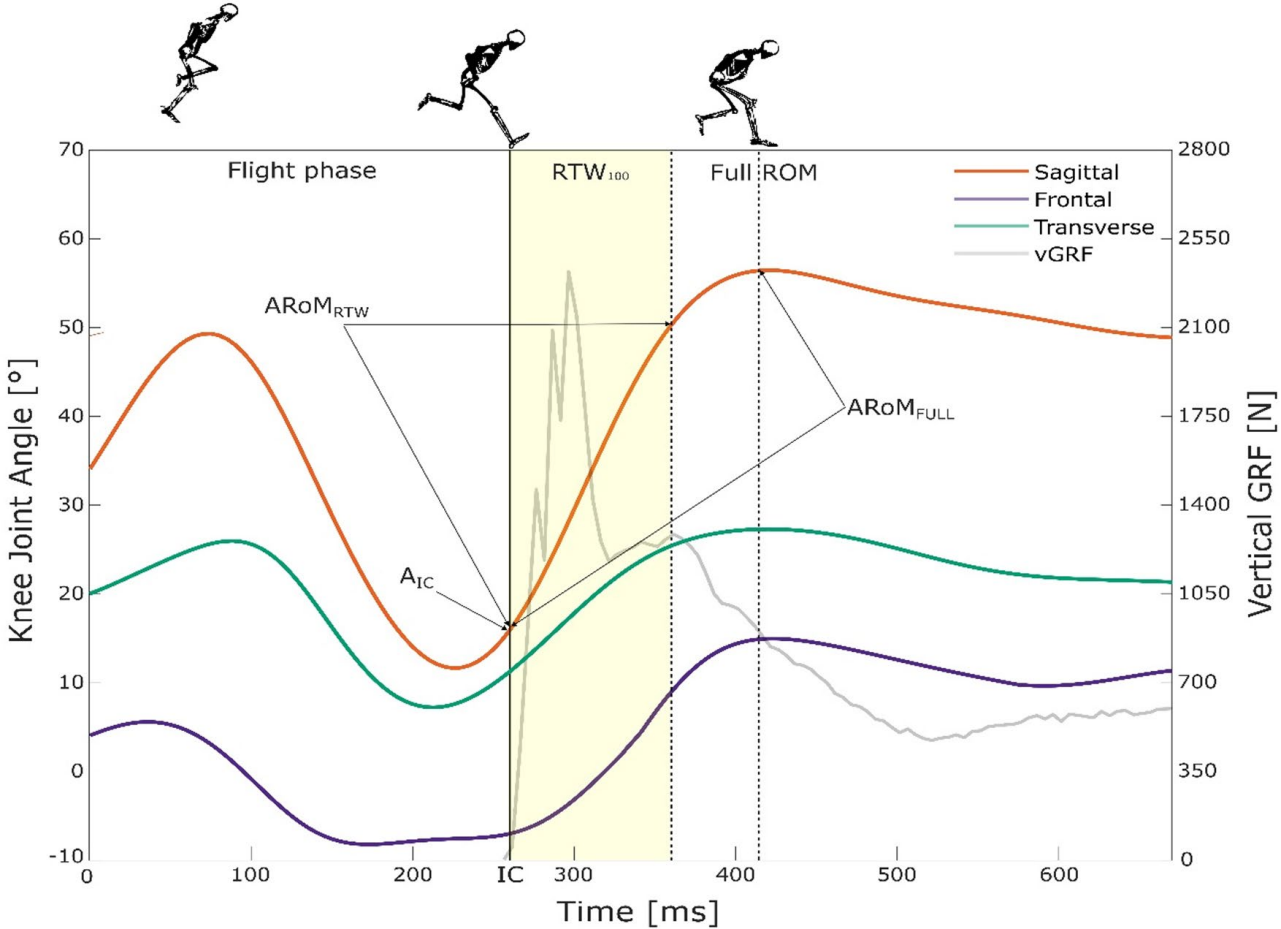
The graph depicts knee kinematics curves in the sagittal (orange line), frontal (violet line) and transverse (green line) planes, along with the vertical ground reaction force (grey line) during Single Leg Hop (SLH). The instant of the Initial Contact (IC), 100 milliseconds after IC (RTW100), and the point of maximum knee flexion (Full ROM) together with the angle at IC and the two considered ROMs are also reported.

Kinematic and dynamic data were initially preprocessed using Nexus (Vicon Motion Systems Ltd, Oxford, UK) and then processed in MATLAB (MathWorks, Natick, MA, USA) using custom-developed scripts. Marker positions were filtered through a zero-lag 2nd-order Butterworth low-pass filter with a cut-off frequency identified by a residual analysis on each marker coordinate^44^. Anatomical reference frames were defined from the marker trajectories for both femur and tibia body segments and the 3D knee joint kinematics was extracted according to the ISB guidelines^45^. From the obtained curves, the following parameters were extracted from knee abduction/adduction, flexion/extension, and intra/extra-rotation angles: angle at initial contact (A_IC_); angular RoM in a time window spanning from IC to the maximum knee flexion (ARoM_FULL_); angular RoM within the RTW, i.e. from IC to 100 ms after IC (ARoM_RTW_) (Fig. 2). Briefly, A_IC_ represented the knee joint angle at the instant the participant contacts the force platform, while ARoM_FULL_ and ARoM_RTW_ were obtained as the difference between the maximum and the minimum angles in given time window^28^. In particular, ARoM_FULL_ referred to the minimum-maximum angle difference observed in each plane of motion from IC up to the instant at which maximum knee flexion angle after landing was reached. This time window was selected based on the consideration that the risk of ACL injury beyond this point can be considered negligible. Differently, ARoM_RTW_ was calculated as the minimum-maximum difference within a defined time window corresponding to the first 100 ms after IC, for all three planes, regardless of whether maximum joint excursion was reached in the sagittal plane. With respect to frontal plane kinematics, knee abduction (also referred to as knee valgus angle) describes the angle at the knee where the tibia/shank moves away from the body midline in the frontal plane and relative to the femur’s long axis. In the transverse plane, knee external rotation angle is defined as the angle resulting from the tibia rotating externally with respect to the femur^10^.

### Statistical analysis

For each parameter, a preliminary outlier analysis was performed based on a threshold of ± 3 on the z-score to remove outliers from the dataset^46^. After outliers removal, Shapiro-Wilk test was conducted to investigate the normality of data distribution. To better characterize the sample data, a preliminary analysis was performed to explore between-limb differences within each population (R_L_/U_L_ for ACLR; ND_L_/D_L_ for HC). In particular, paired t-test or Wilcoxon signed ranks test was carried out for each parameter depending on whether variable distribution was normal or not normal, respectively.

A correlation analysis was then performed to investigate the relationship between the obtained angular variables. Specifically, the Pearson’s or Spearman’s correlation coefficient between each pair of A_IC_, ARoM_FULL_ and ARoM_RTW_ was calculated for each plane (sagittal, frontal, transverse), group and limb (R_L_ and U_L_ for ACLR; ND_L_ and D_L_ for HC), depending on data distribution. The correlation between the parameters was classified as: negligible (absolute correlation coefficient: 0-0.29), low (0.3–0.49), moderate (0.5–0.69), high (0.7–0.89), and very high (0.9-1.00)^47^. R^2^ was selected as the effect size measure for all statistical tests, with values of 0.01, 0.09, and 0.25 indicating thresholds for small, medium, and large effect sizes, respectively^48^. Statistical analysis was performed using the GraphPad software (version 8.4.2, California, USA, α = 0.05).

## Results

### Between-limb differences

Table 1 reports the results obtained from the between-limb comparison analysis. In SLCDL, significant differences were reported in ACLR when observing ARoM_FULL_ and ARoM_RTW_ in the sagittal plane. In particular, lower values were found in R_L_ compared to U_L_ for both ARoM_FULL_ (mean difference: -7 ± 5°; t: 3.574, *p*: 0.012, R^2^: 0.70) and ARoM_RTW_ (-5 ± 4°; t: 3.766, *p*: 0.004, R^2^: 0.61). Significant differences were also reported in HC, where ND_L_ reported slightly lower ARoM_RTW_ than D_L_ in both sagittal (-2 ± 4°; t: 2.259, *p*: 0.037, R^2^: 0.28) and transverse plane (-1 ± 3°; t: 2.384, *p*: 0.028, R^2^: 0.23).

**Table 1:** Mean (standard deviation) of angular variables ARoM_FULL_, ARoM_RTW_, and A_IC_ in the three planes of motion during the Single Leg Cross Drop Landing (SLCDL) and Single Leg Hop (SLH) tasks. Values are reported for reconstructed (R_L_) and uninvolved (U_L_) limbs in ACLR, and non-dominant (ND_L_) and dominant (D_L_) limbs in HC. Significant differences between limbs are highlighted in bold (*p* < 0.05).

	SLCDL				SLH			
	ACLR		HC		ACLR		HC	
	*R*_L_	U_L_	ND_L_	D_L_	*R*_L_	U_L_	ND_L_	D_L_
**SAGITTAL**								
ARoM_FULL_ (°)	**37 (7)**	**44 (7)**	42 (6)	44 (6)	**40 (8)**	**46 (12)**	40 (6)	44 (8)
ARoM_RTW_ (°)	**27 (5)**	**33 (4)**	**31 (4)**	**33 (3)**	**32 (5)**	**36 (4)**	**33 (4)**	**36 (4)**
A_IC_ (°)	9 (7)	12 (7)	11 (7)	13 (5)	**11 (6)**	**16 (8)**	12 (6)	12 (5)
**FRONTAL**								
ARoM_FULL_ (°)	8 (5)	7 (6)	9 (5)	10 (4)	9 (6)	5 (4)	**7 (5)**	**9 (5)**
ARoM_RTW_ (°)	5 (3)	5 (3)	7 (3)	8 (3)	6 (5)	4 (3)	**6 (4)**	**8 (4)**
A_IC_ (°)	4 (6)	3 (3)	4 (4)	4 (3)	4 (4)	5 (4)	5 (3)	5 (4)
**TRANSVERSE**								
ARoM_FULL_ (°)	9 (5)	7 (4)	10 (6)	11 (5)	11 (5)	8 (7)	12 (7)	13 (7)
ARoM_RTW_ (°)	6 (4)	4 (3)	**6 (4)**	**8 (3)**	7 (3)	6 (6)	9 (5)	10 (5)
A_IC_ (°)	3 (6)	2 (5)	3 (5)	3 (5)	0 (7)	− 2 (7)	2 (6)	0 (6)

In SLH, significant between-limb differences were found in ACLR in the sagittal plane only, with lower values in R_L_ compared to U_L_ in all three parameters (ARoM_FULL_: -4 ± 4°; t: 2.737, *p*: 0.026, R^2^: 0.48; ARoM_RTW_: -3 ± 4°; t: 2.934, *p*: 0.017, R^2^: 0.49; A_IC_: -4 ± 6°; t: 2.366, *p*: 0.04, R^2^: 0.38). Significant differences were also found in HC, with lower angles in ND_L_ than D_L_ in sagittal ARoM_RTW_ (-3 ± 5°; t: 2.394; *p*: 0.027; R^2^: 0.23) and both frontal plane RoMs (ARoM_FULL_: -1 ± 3°; t: 2.102, *p*: 0.049, R^2^: 0.19; and ARoM_RTW_: -2 ± 3°; t: 2.293, *p*: 0.033, R^2^: 0.22).

### Correlation analysis

**Fig. 3:**
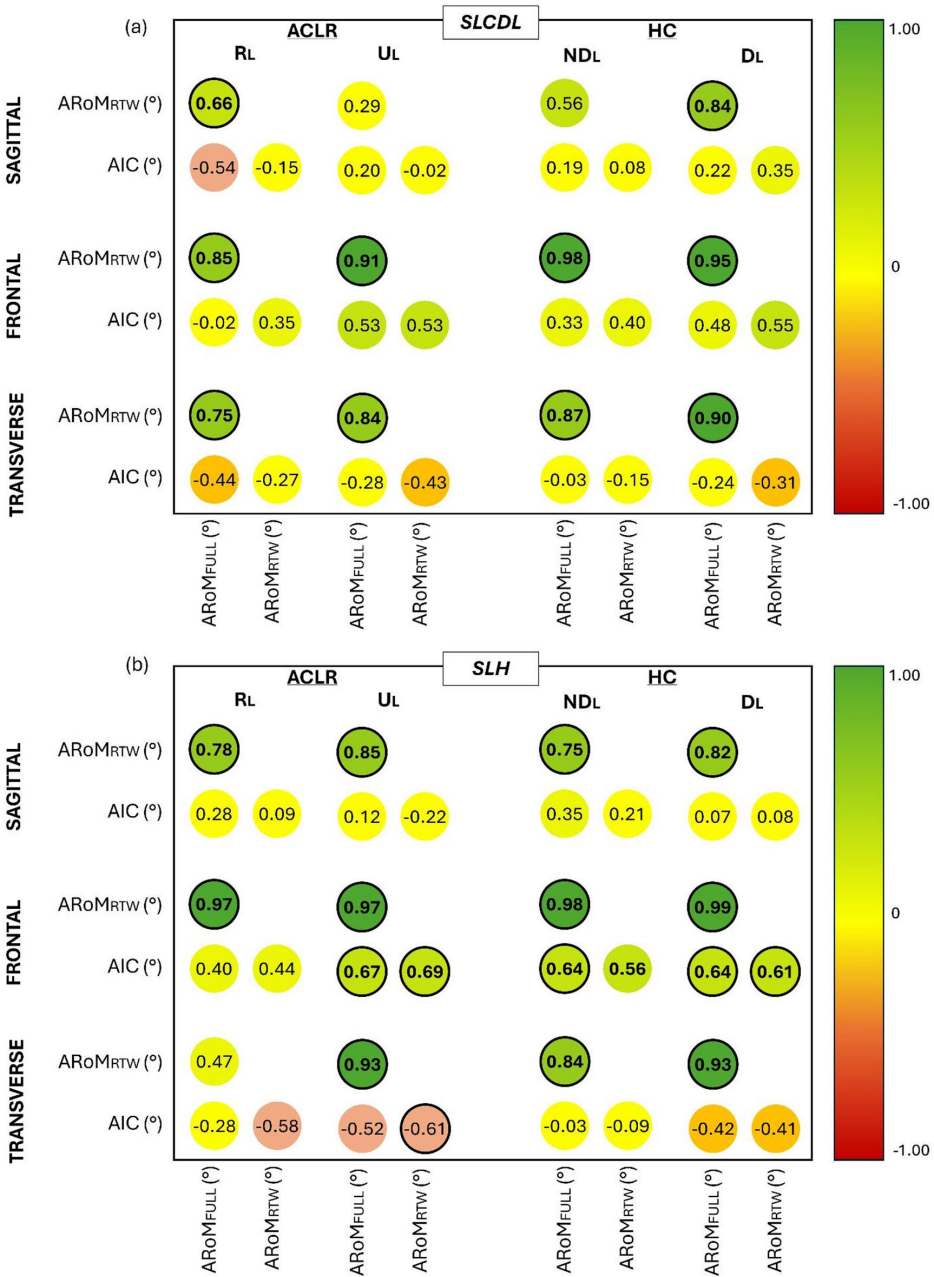
Results from correlation analysis between RoMs and A_IC_ for SLCDL (**a**) and SLH (**b**). Correlations values were divided into very high positive (r: 0.9 to 1.0; dark green); high positive (r: 0.7 to 0.89; green); moderate positive (r: 0.5 to 0.69; light green); low positive (r: 0.3 to 0.49; pea green); negligible (r: 0 to 0.29 and r: 0 to -0.29; yellow); low negative (r: -0.3 to -0.49; orange); moderate negative (r: -0.5 to -0.69; light red); high negative (r: -0.7 to -0.89; red); very high negative (r: -0.9 to -1.0; dark red). Significant and above-critical threshoopèld (r = ± 0.60) coefficients are in bolded circles.

**Fig. 4:**
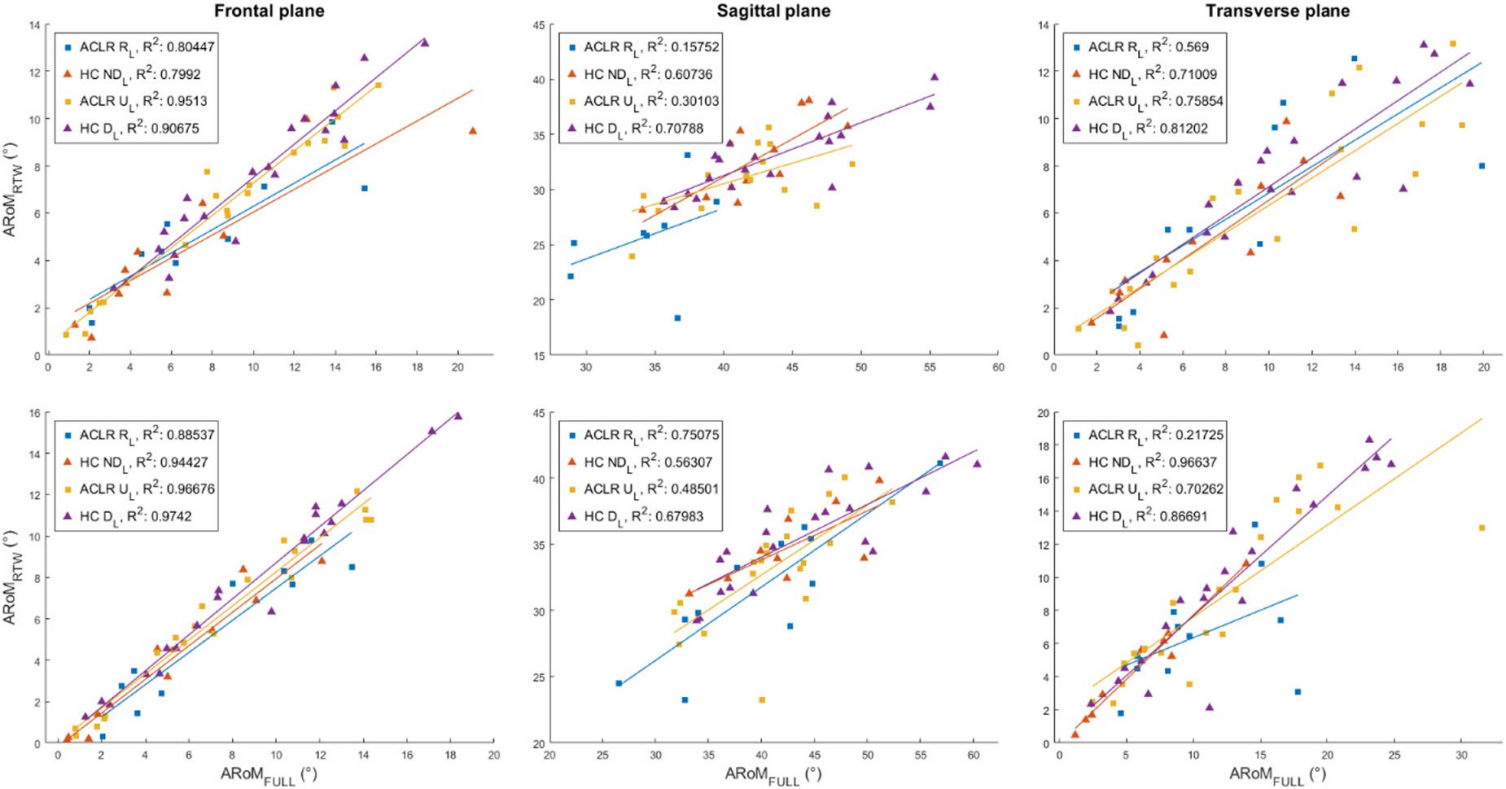
Scatter plots with linear trending lines indicating the relationships per each limb and group between ARoM_FULL_ (X axis) and ARoM_RTW_ (Y axis) in the frontal (knee abduction), sagittal (knee flexion) and transverse planes (knee internal rotation), for both SLCDL (top row) and SLH (bottom row) (*p* < 0.05).

Figure 3a-b display the results obtained from the correlation analysis between RoMs (ARoM_FULL_ and ARoM_RTW_) and A_IC_. Most of the correlations between ARoM_FULL_ and ARoM_RTW_ were moderate-to-very-high positive correlations (r: 0.56–0.99; *p* < 0.05; R^2^: 0.31–0.98) in both groups, limbs and motor tasks and across all planes (Fig. 4). In SLCDL, non-significant low correlations between ARoM_FULL_ and ARoM_RTW_ were only found in the sagittal plane for U_L_ in ACLR and ND_L_ in HC (*p* > 0.05). In SLH, non-significant correlations between ARoM_FULL_ and ARoM_RTW_ were exclusively observed in the transverse plane for R_L_ in ACLR (*p >* 0.05).

When assessing the relations between A_IC_ and ARoM_FULL_ in SLH, moderate statistically significant positive correlations were only found in the frontal plane for U_L_ of ACLR (r: 0.67; *p*: 0.034; R^2^: 0.45), and both D_L_ (r: 0.64; *p*: 0.002; R^2^: 0.41) and ND_L_ (r: 0.64; *p*: 0.002; R^2^: 0.41) of HC. When considering the relationship between A_IC_ and ARoM_RTW_ in SLH, moderate significant positive correlations were found in the frontal plane for U_L_ of ACLR (r: 0.69; *p*: 0.029; R^2^: 0.48) and D_L_ of HC (r: 0.61; *p*: 0.004; R^2^: 0.17).

In general, slightly higher significant correlations between ARoM_FULL_ and A_IC_ were found in SLH (mean r value: 0.65; *p* < 0.05; mean R^2^: 0.42) than in SLCDL (r: 0.48; *p*: 0.031; R^2^: 0.23). Similar considerations can be made for the correlation coefficients between ARoM_RTW_ and A_IC_ (SLH, mean r value: 0.62, *p* < 0.05, R^2^: 0.38; SLCDL, r: 0.55; *p*: 0.012, R^2^: 0.30).

## Discussion

The present study aimed to explore the relationships between knee joint kinematic parameters commonly used in the literature to identify altered biomechanical behaviors associated with ACL injury risk. To this aim, knee A_IC_, as well as ARoM_FULL_ and ARoM_RTW_ were obtained during SLH and SLCDL. Consequently, their relationship was tested through correlation analysis.

We hypothesized that ARoM_FULL_ and ARoM_RTW_ would be highly correlated, whereas A_IC_ was not expected to be necessarily correlated with either RoM measure. A key finding of this study is the strong correlation between ARoM_FULL_ and ARoM_RTW_ in both ACLR and HC groups across both investigated motor tasks. This result supports our first research hypothesis, suggesting that the knee joint kinematics in the first 100 milliseconds after landing is descriptive of the knee kinematics from IC to the peak knee flexion. This reinforces the feasibility of using ARoM_RTW_ as a standardized metric for knee motion assessment in ACL injury research and rehabilitation in patients with ACL reconstruction. Specifically, a practical advantage of using ARoM_RTW_ is that it consistently exhibited lower variability, as reflected by smaller standard deviations compared to ARoM_FULL_. By limiting the analysis to the very early post-landing phase, ARoM_RTW_ reduces the influence of inter-individual and task-dependent differences in landing mechanics that contribute to variability in ARoM_FULL_, thereby providing a more consistent and less condition-dependent parameter. For instance, in drop landing tasks, full knee RoM is influenced by the height of the drop, with higher heights leading to greater knee flexion angles^49,50^. Since different studies employ varying box heights for drop landings (e.g., 15 cm in^23^, 20 cm in^30^, ~ 30 cm in^18,20,24,43^, the adoption of ARoM_RTW_ may help in reducing the effect of these confounding variables, allowing for a standard interpretation of the data across different conditions. However, if the focus of analysis is on the full range of motion after landing, it is crucial to clearly define the anatomical plane of interest and establish precise criteria for determining the end of the time window under consideration. Given that ACL injuries typically occur within the first 100 milliseconds after landing, and peak external loads are observed in this window^14^, ARoM_RTW_ emerges as a meaningful and practical parameter, as it aligns closely with the temporal characteristics of ACL rupture mechanisms. However, its clinical relevance in predicting ACL injury risk relative to other well-established knee kinematic metrics remains to be further investigated^51^.

In contrast, our results indicate that knee A_IC_ and RoM parameters are not necessarily correlated across all three anatomical planes. This finding is in line with our second hypothesis and highlights that A_IC_ alone does not fully describe the knee joint response after landing, thus emphasizing the need for caution when using it alone without RoM measures as an ACL injury risk indicator. This is likely due to the different biomechanical nature of parameters. In fact, A_IC_ reflects the athlete’s preparatory movement strategy before and at the moment of foot contact^52,53^, whereas RoM captures the knee’s dynamic behavior in response to impact forces over given periods of time^54,55^. Although both metrics tend to show similar directional changes (i.e., increases) in response to different landing strategies, such as natural versus soft landings^55^, the low correlation in our study suggests that an athlete’s landing posture at IC may not necessarily dictate subsequent post-impact knee motion. This appears particularly evident in the sagittal plane, where the lowest and non-significant correlation coefficients were observed. Accordingly, using A_IC_ alone provides limited insights into the movement immediately after IC, when higher loads impact the knee joint structures^10,56^.

Notably, few moderate positive correlations between A_IC_ and RoM parameters were found in the frontal plane in both populations and each motor task, especially in the uninvolved limb of ACLR during the SLH task (*r* > 0.6). These correlations in the frontal plane between A_IC_ and RoMs suggest that greater knee abduction angles at IC might be associated with higher valgus collapse. As valgus collapse has been consistently indicated as one of the kinematic patterns associated with ACL injury mechanisms^7–10^, A_IC_ may provide complementary information about frontal-plane movement strategies during high-demand tasks. However, as mentioned above, this relationship is absent when considering the sagittal plane. This finding might reflect a greater ability to adjust and control movement in the sagittal plane after IC, enabling participants to exhibit knee joint RoMs independently of the knee angle at foot contact. Although previous research has reported behavioural associations between prelanding or initial-contact knee kinematics and landing kinetics or knee RoMs in the sagittal plane^55,57^, the present findings suggest that this relationship may not necessarily transfer to a direct correlation between sagittal knee A_IC_ and RoMs. However, further research is warranted to verify this hypothesis. Overall, the present findings provide preliminary evidence that post-landing knee control may be influenced by factors beyond the initial joint position, potentially including neuromuscular strategies and reactive control mechanisms.

It is well known that ACL injury can be facilitated by specific motor behaviors involving landing with the leg almost extended, followed by simultaneous and excessive knee abduction and rotation (valgus collapse)^25^. When comparing between-limb differences, the angular values in both investigated motor tasks are in line with those reported in the literature during similar movements involving a single-leg landing^30^. Specifically, participants with ACL reconstruction consistently showed significantly lower sagittal plane angles in the reconstructed limb compared to the unaffected limb, particularly in ARoM_FULL_ and ARoM_RTW_. In SLCDL, these differences reached on average 7° and 5°, respectively, and were similarly observed in SLH (4° and 3°), along with an additional significant reduction in A_IC_ (4°). While these differences may suggest a tendency toward a stiffer landing strategy in the affected limb, characterized by reduced knee flexion after initial contact, RoM deviations of less than 10° should be interpreted with caution in the context of impaired load absorption and potentially increased stress on the ACL^32,58^. Their clinical relevance remains uncertain, particularly in the context of highly variable individual adaptations post-ACL reconstruction. Similarly, healthy controls also exhibited significant between-limb differences, but these were even smaller in magnitude (1°–3°) and observed primarily in ARoM_RTW_ and in the frontal plane. These subtle asymmetries likely reflect typical variations related to limb dominance and motor preference rather than dysfunctional movement. Although not the primary interest of this work, the observed asymmetries characterize the sample and highlight the relevance of tracking these parameters in the post-surgery period, even after long-term recovery and when athletes are considered ready to return to sport.

It is worth to mention that the aim of the present study was not to determine which parameter is more indicative of increased knee stress and ACL injury risk, but rather to investigate whether RoMs and A_IC_ displayed common trends in terms of knee kinematics. On this aspect, some works have reported that, in the frontal plane, female athletes who reported greater A_IC_ and angular displacements (RoMs) during a drop jump (from IC to Toe-Off) subsequently sustained a primary or secondary ACL tear^18,34^. Considering this result, additional studies focusing on sagittal and transverse planes are needed to determine whether RoMs or A_IC_ is more effective in detecting injury risk conditions, thereby informing future biomechanical analysis.

The present study has some limitations that should be acknowledged. First, although the present work provides a comprehensive analysis in all planes of motion, caution is needed when interpreting results in planes other than the sagittal one, as movements in the frontal and, especially, transverse planes are less pronounced. Nevertheless, the marker protocol adopted in the present study was specifically developed for the analysis of three-dimensional lower limb kinematics, featuring a high number of markers placed around the knee joint and thus allowing to accurately track subtle movements in planes other than the sagittal one^39^. Second, although the current sample size satisfied the requirements of the a priori power analysis, the investigation was restricted to a relatively small group of male soccer players. In addition, ACL-reconstructed athletes underwent different surgical techniques: six athletes received a patellar tendon autograft, whereas five underwent reconstruction with hamstring tendon autograft involving gracilis and semitendinosus tendons. Evidence regarding biomechanical differences in jump landing movement patterns at RTS between athletes reconstructed with different graft types remains inconclusive or does not indicate a consistent injury risk-related trend^59,60^. Examining whether the relationship between knee angle at initial contact and post-landing RoMs differs between graft types, therefore, represents an important direction for future research. Moreover, inclusion of female athletes and participants from other open-skills sports characterized by frequent landing demands, such as basketball or volleyball, would support the generalizability of the current findings. Finally, the present study considered two different hop tasks, namely the SLH and SLCDL. Examining this relationship across different jump tasks and/or sport specific movements – such as change of direction maneuvers – may help to further strengthen the results observed.

## Conclusions

In conclusion, knee ranges of motion within the time window from the initial contact to full knee flexion and from initial contact to the first 100 millisecond during the observed landing tasks were strongly correlated especially in frontal and transverse planes. These results suggest that both ranges of motion provide comparable information related to the movement itself after initial contact and support the use of a time window of 100 milliseconds to facilitate unbiased comparison among different athletes and motor tasks. Conversely, the lack of consistent correlation between the knee angle at initial contact and the knee ranges of motion after initial contact suggests caution when considering these parameters as equally informative about ACL injury risk/recovery indicators. Using the knee angle at initial contact alone does not provide insights into the movement immediately after landing, when higher loads impact the knee joint structures and ACL injury typically occurs. Since ACL injuries typically occur within the first 100 milliseconds post-IC, assessing RoM within this window provides critical insights into knee joint behavior under high-impact loads. Standardizing this metric could enhance injury screening protocols and inform targeted rehabilitation strategies aimed at reducing ACL injury risk and optimizing post-surgical recovery.

## Data Availability

The datasets generated during and/or analyzed during the current study are available from the corresponding author on reasonable request.

## References

[1] Irmischer, B. S. et al. Effects of a knee ligament injury prevention exercise program on impact forces in women. *J. Strength. Cond Res.* **18**, 703–707. 10.1519/R-13473.1 (2004). 15574070 10.1519/R-13473.1

[2] Hewett, T. E., Di Stasi, S. L. & Myer, G. D. Current concepts for injury prevention in athletes after anterior cruciate ligament reconstruction. *Am. J. Sports Med.* **41**, 216–224. 10.1177/0363546512459638 (2013). 23041233 10.1177/0363546512459638 PMC3592333

[3] Paterno, M. V., Rauh, M. J., Schmitt, L. C., Ford, K. R. & Hewett, T. E. Incidence of second ACL injuries 2 years after primary ACL reconstruction and return to sport. *Am. J. Sports Med.* **42**, 1567–1573. 10.1177/0363546514530088 (2014). 24753238 10.1177/0363546514530088 PMC4205204

[4] Bahr, R. & Krosshaug, T. Understanding injury mechanisms: A key component of preventing injuries in sport. *Br. J. Sports Med.* **39**, 324–329. 10.1136/bjsm.2005.018341 (2005). 15911600 10.1136/bjsm.2005.018341 PMC1725226

[5] Gokeler, A. et al. Return to sports after ACL injury 5 years from now: 10 things we must do. *J. Exp. Orthop.* **9**, 111. 10.1186/s40634-022-00514-7 (2022). 36396880 10.1186/s40634-022-00548-x PMC9672288

[6] Griffin, L. Y. et al. Noncontact anterior cruciate ligament injuries: risk factors and prevention strategies. *J. Am. Acad. Orthop. Surg.* **8**, 141–150. 10.5435/00124635-200005000-00001 (2000). 10874221 10.5435/00124635-200005000-00001

[7] Mehl, J. et al. Evidence-based concepts for prevention of knee and ACL injuries. 2017 guidelines of the ligament committee of the German Knee Society (DKG). *Arch. Orthop. Trauma. Surg.* **138**, 51–61. 10.1007/s00402-017-2809-5 (2018). 28983841 10.1007/s00402-017-2809-5

[8] Hewett, T. E., Ford, K. R., Hoogenboom, B. J. & Myer, G. D. Understanding and Preventing ACL Injuries: Current Biomechanical and Epidemiologic Considerations. *N Am. J. Sports Phys. Ther.* **5**, 234–251 (2010). 21655382 PMC3096145

[9] Weiss, K. & Whatman, C. Biomechanics Associated with Patellofemoral Pain and ACL Injuries in Sports. *Sports Med.* **45**, 1325–1337. 10.1007/s40279-015-0353-4 (2015). 26130304 10.1007/s40279-015-0353-4

[10] Shimokochi, Y. & Shultz, S. J. Mechanisms of Noncontact Anterior Cruciate Ligament Injury. *J. Athl Train.* **43**, 396–408. 10.4085/1062-6050-43.4.396 (2008). 18668173 10.4085/1062-6050-43.4.396 PMC2474820

[11] Della Villa, F. et al. Systematic video analysis of ACL injuries in professional male football (soccer): injury mechanisms, situational patterns and biomechanics study on 134 consecutive cases. *Br. J. Sports Med.* **54**, 1423–1432. 10.1136/bjsports-2019-101247 (2020). 32561515 10.1136/bjsports-2019-101247

[12] Della Villa, F. et al. Systematic Video Analysis of Anterior Cruciate Ligament Injuries in Professional Male Rugby Players: Pattern, Injury Mechanism, and Biomechanics in 57 Consecutive Cases. *Orthop. J. Sports Med.* **9**, 23259671211048182. 10.1177/23259671211048182 (2021). 34805419 10.1177/23259671211048182 PMC8597070

[13] Koga, H., Nakamae, A., Shima, Y., Bahr, R. & Krosshaug, T. Hip and Ankle Kinematics in Noncontact Anterior Cruciate Ligament Injury Situations: Video Analysis Using Model-Based Image Matching. *Am. J. Sports Med.* **46**, 333–340. 10.1177/0363546517732750 (2018). 29024605 10.1177/0363546517732750

[14] Stephenson, M. L. et al. Effects of timing of signal indicating jump directions on knee biomechanics in jump-landing-jump tasks. *Sports Biomech.* **17**, 67–82. 10.1080/14763141.2017.1346141 (2018). 28730871 10.1080/14763141.2017.1346141

[15] Sigurðsson, H. B., Karlsson, J., Snyder-Mackler, L. & Briem, K. Kinematics observed during ACL injury are associated with large early peak knee abduction moments during a change of direction task in healthy adolescents. *J. Orthop. Res.* **39**, 2281–2290. 10.1002/jor.24942 (2021). 33280158 10.1002/jor.24942 PMC8179932

[16] Laughlin, W. A. et al. The effects of single-leg landing technique on ACL loading. *J. Biomech.* **44**, 1845–1851. 10.1016/j.jbiomech.2011.04.010 (2011). 21561623 10.1016/j.jbiomech.2011.04.010

[17] Kernozek, T. W. & Ragan, R. J. Estimation of anterior cruciate ligament tension from inverse dynamics data and electromyography in females during drop landing. *Clin. Biomech.* **23**, 1279–1286. 10.1016/j.clinbiomech.2008.08.001 (2008). 10.1016/j.clinbiomech.2008.08.001 18790553

[18] Hewett, T. E. et al. Biomechanical Measures of Neuromuscular Control and Valgus Loading of the Knee Predict Anterior Cruciate Ligament Injury Risk in Female Athletes: A Prospective Study. *Am. J. Sports Med.* **33**, 492–501. 10.1177/0363546504269591 (2005). 15722287 10.1177/0363546504269591

[19] DiCesare, C. A., Kiefer, A. W., Bonnette, S. & Myer, G. D. High-Risk Lower-Extremity Biomechanics Evaluated in Simulated Soccer-Specific Virtual Environments. *J. Sport Rehabil*. **29**, 294–300. 10.1123/jsr.2018-0237 (2020). 30676190 10.1123/jsr.2018-0237 PMC9892798

[20] Leppänen, M. et al. Stiff Landings Are Associated with Increased ACL Injury Risk in Young Female Basketball and Floorball Players. *Am. J. Sports Med.* **45**, 386–393. 10.1177/0363546516665810 (2017). 27637264 10.1177/0363546516665810

[21] McLean, S. G. et al. Evaluation of a two dimensional analysis method as a screening and evaluation tool for anterior cruciate ligament injury. *Br. J. Sports Med.* **39**, 355–362. 10.1136/bjsm.2005.018598 (2005). 15911607 10.1136/bjsm.2005.018598 PMC1725240

[22] Gokeler, A., Dingenen, B. & Hewett, T. E. Rehabilitation and Return to Sport Testing After Anterior Cruciate Ligament Reconstruction: Where Are We in 2022? *Arthrosc. Sports Med. Rehabil*. **4**, e77–e82. 10.1016/j.asmr.2021.10.025 (2022). 35141539 10.1016/j.asmr.2021.10.025 PMC8811523

[23] Kotsifaki, A. et al. Single leg vertical jump performance identifies knee function deficits at return to sport after ACL reconstruction in male athletes. *Br. J. Sports Med.* **56**, 490–498. 10.1136/bjsports-2021-104692 (2022). 35135826 10.1136/bjsports-2021-104692 PMC9016240

[24] Morishige, Y. et al. Difference in leg asymmetry between female collegiate athletes and recreational athletes during drop vertical jump. *J. Orthop. Surg. Res.* **14**, 424. 10.1186/s13018-019-1490-5 (2019). 31822295 10.1186/s13018-019-1490-5 PMC6905029

[25] Paterno, M. V. et al. Biomechanical measures during landing and postural stability predict second anterior cruciate ligament injury after anterior cruciate ligament reconstruction and return to sport. *Am. J. Sports Med.* **38**, 1968–1978. 10.1177/0363546510376053 (2010). 20702858 10.1177/0363546510376053 PMC4920967

[26] Alarifi, S. M. et al. Biomechanical Analysis After Anterior Cruciate Ligament Reconstruction at the Return-to-Sport Time Point. *Orthop. J. Sports Med.* **13**, 23259671251340302. 10.1177/23259671251340302 (2025). 40438185 10.1177/23259671251340302 PMC12117235

[27] Kotsifaki, A. et al. Single leg hop for distance symmetry masks lower limb biomechanics: time to discuss hop distance as decision criterion for return to sport after ACL reconstruction? *Br. J. Sports Med.* **56**, 249–256. 10.1136/bjsports-2020-103677 (2022). 33687928 10.1136/bjsports-2020-103677

[28] Gokeler, A. et al. Abnormal landing strategies after ACL reconstruction. *Scand. J. Med. Sci. Sports*. **20**, e12–e19. 10.1111/j.1600-0838.2008.00873.x (2010). 19210671 10.1111/j.1600-0838.2008.00873.x

[29] Oberländer, K. D., Brüggemann, G. P., Höher, J. & Karamanidis, K. Altered landing mechanics in ACL-reconstructed patients. *Med. Sci. Sports Exerc.* **45**, 506–513. 10.1249/MSS.0b013e3182752ae3 (2013). 23034645 10.1249/MSS.0b013e3182752ae3

[30] Rocchi, J. E. et al. Timing of muscle activation is altered during single-leg landing tasks after anterior cruciate ligament reconstruction at the time of return to sport. *Clin. J. Sport Med.* **30**, e186–e193. 10.1097/JSM.0000000000000659 (2020). 30418218 10.1097/JSM.0000000000000659

[31] Kotsifaki, A., Korakakis, V., Whiteley, R., Van Rossom, S. & Jonkers, I. Measuring only hop distance during single leg hop testing is insufficient to detect deficits in knee function after ACL reconstruction: A systematic review and meta-analysis. *Br. J. Sports Med.* **54**, 139–153. 10.1136/bjsports-2018-099918 (2020). 31142471 10.1136/bjsports-2018-099918

[32] Larwa, J., Stoy, C., Chafetz, R. S., Boniello, M. & Franklin, C. Stiff landings, core stability, and dynamic knee valgus: A systematic review on documented anterior cruciate ligament ruptures in male and female athletes. *Int. J. Environ. Res. Public. Health*. **18**, 3826. 10.3390/ijerph18073826 (2021). 33917488 10.3390/ijerph18073826 PMC8038785

[33] Montgomery, C. et al. Mechanisms of ACL injury in professional rugby union: A systematic video analysis of 36 cases. *Br. J. Sports Med.* **52**, 994–1001. 10.1136/bjsports-2016-096425 (2018). 28039125 10.1136/bjsports-2016-096425

[34] Bates, N. A., Myer, G. D., Hale, R. F., Schilaty, N. D. & Hewett, T. E. Prospective Frontal Plane Angles Used to Predict ACL Strain and Identify Those at High Risk for Sports-Related ACL Injury. *Orthop. J. Sports Med.* **8**, 2325967120957646. 10.1177/2325967120957646 (2020). 33110927 10.1177/2325967120957646 PMC7557696

[35] Nagai, T., Sell, T. C., House, A. J., Abt, J. P. & Lephart, S. M. Knee proprioception and strength and landing kinematics during a single-leg stop-jump task. *J. Athl Train.* **48**, 31–38. 10.4085/1062-6050-48.1.14 (2013). 23672323 10.4085/1062-6050-48.1.14 PMC3554030

[36] Tegner, Y. & Lysholm, J. Rating Systems in the Evaluation of Knee Ligament Injuries. *Clin. Orthop. Relat. Res.* **198**, 43–49 (1985). 4028566

[37] van Melick, N., Meddeler, B. M., Hoogeboom, T. J., Nijhuis-van der Sanden, M. W. G. & van Cingel R.E.H. How to determine leg dominance: The agreement between self-reported and observed performance in healthy adults. *PLoS One*. **12**, e0189876. 10.1371/journal.pone.0189876 (2017). 29287067 10.1371/journal.pone.0189876 PMC5747428

[38] DeLang, M. D., Rouissi, M., Braggazi, N. L., Chamari, K. & Salamh, P. A. Soccer footedness and between-limb muscle strength: Systematic review and meta-analysis. *Int. J. Sports Physiol. Perform.* **14**, 551–562. 10.1123/ijspp.2018-0336 (2019). 30975008 10.1123/ijspp.2018-0336

[39] Pillet, H., Bonnet, X., Lavaste, F. & Skalli, W. Evaluation of force plate-less estimation of the trajectory of the centre of pressure during gait. Comparison of two anthropometric models. *Gait Posture*. **31**, 147–152. 10.1016/j.gaitpost.2009.09.014 (2010). 19864138 10.1016/j.gaitpost.2009.09.014

[40] Smeets, A. et al. Is knee neuromuscular activity related to anterior cruciate ligament injury risk? A pilot study. *Knee* **26**, 40–51. 10.1016/j.knee.2018.10.006 (2019). 30415973 10.1016/j.knee.2018.10.006

[41] Webster, K. E., Ristanis, S. & Feller, J. A. A longitudinal investigation of landing biomechanics following anterior cruciate ligament reconstruction. *Phys. Ther. Sport*. **50**, 36–41. 10.1016/j.ptsp.2021.03.012 (2021). 33865216 10.1016/j.ptsp.2021.03.012

[42] Gustavsson, A. et al. A test battery for evaluating hop performance in patients with an ACL injury and patients who have undergone ACL reconstruction. *Knee Surg. Sports Traumatol. Arthrosc.* **14**, 778–788. 10.1007/s00167-006-0045-6 (2006). 16525796 10.1007/s00167-006-0045-6

[43] DiCesare, C. A. et al. Reliability of 3-Dimensional Measures of Single-Leg Cross Drop Landing Across 3 Different Institutions: Implications for Multicenter Biomechanical and Epidemiological Research on ACL Injury Prevention. *Orthop. J. Sports Med.* **3**, 2325967115617905. 10.1177/2325967115617905 (2015). 26779550 10.1177/2325967115617905 PMC4710117

[44] Winter, D. A. & Biomechanics in *Biomechanics of human movement*. (ed. John Wiley & Sons Inc) 65–83 (1979).

[45] Wu, G. et al. ISB recommendation on definitions of joint coordinate system of various joints for the reporting of human joint motion part I ankle, hip, and spine. *J. Biomech.* **35**, 543–548. 10.1016/s0021-9290(01)00222-6 (2022). 10.1016/s0021-9290(01)00222-6 11934426

[46] Pfeiffer, S. J. et al. Association of jump-landing biomechanics with tibiofemoral articular cartilage composition 12 months after ACL reconstruction. *Orthop. J. Sports Med.* **9**, 23259671211016424. 10.1177/23259671211016424 (2021). 34368382 10.1177/23259671211016424 PMC8299897

[47] Mukaka, M. & Statistics Corner A guide to appropriate use of Correlation coefficient in medical research. *Malawi Med. J.* **24**, 69–71 (2012). www.mmj.medcol.mw 23638278 PMC3576830

[48] Cohen, J. *Statistical Power Analysis for the Behavioral Sciences (Second Edition)* (1988).

[49] Dickin, D. C., Johann, E., Wang, H. & Popp, J. K. Combined effects of drop height and fatigue on landing mechanics in active females. *J. Appl. Biomech.* **31**, 237–243. 10.1123/jab.2014-0190 (2015). 25780957 10.1123/jab.2014-0190

[50] Ali, N., Robertson, D. G. E. & Rouhi, G. Sagittal plane body kinematics and kinetics during single-leg landing from increasing vertical heights and horizontal distances: Implications for risk of non-contact ACL injury. *Knee* **21**, 38–46. 10.1016/j.knee.2012.12.003 (2014). 23274067 10.1016/j.knee.2012.12.003

[51] Belkhelladi, M., Cierson, T. & Martineau, P. A. Biomechanical Risk Factors for Increased Anterior Cruciate Ligament Loading and Injury: A Systematic Review. *Orthop. J. Sports Med.* **13**, 23259671241312681. 10.1177/23259671241312681 (2025). 39958696 10.1177/23259671241312681 PMC11826863

[52] Davis, D. J., Hinshaw, T. J., Critchley, M. L. & Dai, B. Mid-flight trunk flexion and extension altered segment and lower extremity joint movements and subsequent landing mechanics. *J. Sci. Med. Sport*. **22**, 955–961. 10.1016/j.jsams.2019.03.001 (2019). 30902539 10.1016/j.jsams.2019.03.001

[53] Song, Y. et al. Influence of combined posterior and medial-lateral mid-air trunk perturbations on knee biomechanics during single-leg landing. *Front. Sports Act. Living*. **7**, 1697893. 10.3389/fspor.2025.1697893 (2025). 41256235 10.3389/fspor.2025.1697893 PMC12620482

[54] Dai, B. et al. The effects of 2 landing techniques on knee kinematics, kinetics, and performance during stop-jump and side-cutting tasks. *Am. J. Sports Med.* **43**, 466–474. 10.1177/0363546514555322 (2015). 25367015 10.1177/0363546514555322

[55] Li, L. et al. Falling as a strategy to decrease knee loading during landings: Implications for ACL injury prevention. *J. Biomech.* **109**, 109906. 10.1016/j.jbiomech.2020.109906 (2020). 32807342 10.1016/j.jbiomech.2020.109906 PMC7438605

[56] Koga, H. et al. Mechanisms for noncontact anterior cruciate ligament injuries: Knee joint kinematics in 10 injury situations from female team handball and basketball. *Am. J. Sports Med.* **38**, 2218–2225. 10.1177/0363546510373570 (2010). 20595545 10.1177/0363546510373570

[57] Li, L., Song, Y., Jenkins, M. & Dai, B. Prelanding knee kinematics and landing kinetics during single-leg and double-leg landings in male and female recreational athletes. *J. Appl. Biomech.* **39**, 34–41. 10.1123/jab.2022-0147 (2023). 36649716 10.1123/jab.2022-0147

[58] Romanchuk, N. J., Bel, D., Benoit, D. L. & M. J., & Sex-specific landing biomechanics and energy absorption during unanticipated single-leg drop-jumps in adolescents: implications for knee injury mechanics. *J. Biomech.* **113**, 110064. 10.1016/j.jbiomech.2020.110064 (2020). 33190054 10.1016/j.jbiomech.2020.110064

[59] Andrade, D., Fonseca, P., Sousa, F. & Gutierres, M. Does Anterior Cruciate Ligament Reconstruction with a Hamstring Tendon Autograft Predispose to a Knee Valgus Alignment on Initial Contact during Landing? A Drop Vertical Jump Movement Analysis. *Appl. Sci.* **13**, 7363. 10.3390/app13137363 (2023).

[60] Costa, A. et al. Comparative Kinematic Analysis of Patellar vs. Hamstring Autografts in ACL Reconstruction on Side-Hop Test Performance. *Appl. Sci.* **15**, 5569. 10.3390/app15105569 (2025).

